# If there is not one cure for schizophrenia, there may be many

**DOI:** 10.1038/s41537-020-0101-0

**Published:** 2020-04-20

**Authors:** Carina Seah, Kristen J. Brennand

**Affiliations:** 1 Graduate School of Biomedical Science, Icahn School of Medicine at Mount Sinai, New York, NY 10029 USA; 2 Nash Family Department of Neuroscience, Icahn School of Medicine at Mount Sinai, New York, NY 10029 USA; 3 Friedman Brain Institute, Icahn School of Medicine at Mount Sinai, New York, NY 10029 USA; 4 Department of Genetics and Genomics, Icahn School of Medicine at Mount Sinai, New York, NY 10029 USA; 5 Icahn Institute of Genomics and Multiscale Biology, Icahn School of Medicine at Mount Sinai, New York, NY 10029 USA; 6 Black Family Stem Cell Institute, Icahn School of Medicine at Mount Sinai, New York, NY 10029 USA

**Keywords:** Cellular neuroscience, Schizophrenia

The diverse presentation of symptoms experienced by patients with schizophrenia is matched only by the complexity of the genetic architecture that underlies disease risk. To date, hundreds of rare^1,2,3^ and common^4,5^ genetic variants have been associated with schizophrenia. Growing evidence supports the link between genotype and patient outcome, portending a future of precision medicine in psychiatry.

Clinicians still engage in a slow trial-and-error process with each patient, in order to identify the most efficacious drug(s) with the fewest side effects; critically, 30% of cases show little response to first-line antipsychotics^6^. No genetic test can yet match patients to the most appropriate drug; however, patients with rare disruptive variants in gene targets of antipsychotics^7^ and/or higher schizophrenia polygenic risk scores (PRS)^8^ tend to show less improvement with antipsychotic drug treatment. Although all current antipsychotics are antagonists of dopamine receptor signaling, and genetic variation at the dopamine D_2_ receptor may influence response to antipsychotic medications, the modest effect sizes at this locus have to date proven to be of limited clinical utility^9^. More promising, it is already possible to apply genetic markers to predict some adverse metabolic responses to antipsychotic treatment^10^.

We foresee a not-too-distant future whereby a combination of patient genotype, brain imaging and biomarkers efficiently pairs patients to their ideal drug. Already it is possible to apply structural or functional brain measures to stratify patients based on the severity of disease and/or drug response^11–13^. Our expectation is that the predictive power of these circuit-based approaches will be further improved by considering patient genetics^7,8^ or imputed cell-type-specific gene expression^14,15^. An emerging platform involves reprogramming of patient somatic cells to human induced pluripotent stem cells (hiPSCs), in order to empirically test pharmacological response of patient-derived brain cells in vitro^16^. Even without understanding the complex interactions between multifaceted risk variants underlying each patient’s disease risk, researchers can systematically evaluate drug response in a donor and cell-type-specific manner. We hope that patient-specific phenotypic and/or transcriptomic drug screens will uncover novel drugs that act through mechanisms independent of dopamine biology. Most importantly, with an improved ability to predict which subset of patients will respond to which novel treatments, a successful drug need not be efficacious in all patients in order to be clinically useful.

Beyond drug response, hiPSC-based models allow for both targeted and large-scale functional validation of the growing list of variants thought to interact to contribute to schizophrenia risk. Rather than continuing to study disease risk variants one-at-a-time, CRISPR-based technologies can be applied to conduct forward genetic screens, simultaneously assessing the phenotypic impact of hundreds of genes in a single experiment. It is now possible to undertake large-scale studies of risk genes on neuronal gene expression and function, in either pooled or arrayed formats^17^. Key candidate genes identified through such unbiased approaches can hint at points of convergence underlying schizophrenia risk that might represent novel therapeutic targets, or help to explain independent genetic mechanisms leading to disease. Resolving the causal variants underlying schizophrenia and treatment response should dramatically improve our ability to match patients to more effective treatments.

The complex heterogeneity of schizophrenia means that a single cure may not be found. New strategies to stratify etiologically complex patients, diagnosing high-risk individuals prior to psychosis onset, makes possible a future whereby we prevent, rather than treat, schizophrenia. Ultimately, tailoring interventions for each patient achieves the potential of precision medicine, treating schizophrenia with not one silver-bullet drug, but many.
